# Combining Techniques to Treat Isolated Deep Recession-Type Defects: A Case Report with Long-Term Stability

**DOI:** 10.1155/2021/9989281

**Published:** 2021-06-18

**Authors:** João Carnio, Anna Tereza Carnio

**Affiliations:** ^1^Private Practice, Londrina, Parana, Brazil; ^2^University of North Parana, Londrina, Parana, Brazil

## Abstract

**Introduction:**

The purpose of this case report was to show the clinical long-term stability of a successful two-step root coverage procedure. A combination of two single techniques was used to treat an isolated deep-wide defect. *Case Presentation*. A 28-year-old female patient was referred in order to treat a single recession defect at #22. Due to her fear of dental procedures and a poor economic situation, the team developed an alternative solution. They used a modified apically repositioned flap (MARF) to increase the donor area and then a laterally positioned flap (LPF) to treat the root defect. Clinical evaluation at the three-year follow-up revealed complete resolution of the defect, a gain in clinical attachment, excellent esthetic results, and minor morbidity to the patient.

**Conclusion:**

The combination of the MARF and the LPF procedures was able to successfully treat a single deep recession defect with some advantages over traditional techniques such as simplicity, ideal color match of tissues, and the absence of palatal donor tissue.

## 1. Introduction

Periodontal plastic surgery is frequently used to maintain functional dentition and improve patient esthetics. Among various mucogingival deformities, the treatment of root recession is one of the most discussed. An autogenous subepithelial connective tissue graft (CTG) [1] is considered to be the gold standard treatment procedure. However, the wound created in the palate has been associated with a high possibility of bleeding and generates great postoperative discomfort. Due to these inconveniences, other options such as autologous and xenograft materials are available [2], but some patients have refused these treatment options due to the additional cost. When a treatment plan is developed, it is recommended that an assessment of the patient's potential postsurgical pain and discomfort, the esthetic outcome, patient satisfaction, and the overall cost-effectiveness be included [3]. Another point that should be considered is that some patients experience dental fear. If that is the case, a simpler and less invasive surgical intervention should increase patient acceptance [4].

This case report describes a simple alternative for the treatment of a deep and wide single recession [5] utilizing a two-step procedure that did not employ the palatal donor source, promoted less morbidity, and improved postoperative comfort, with excellent esthetic results.

## 2. Case Presentation

**Figure 1:**
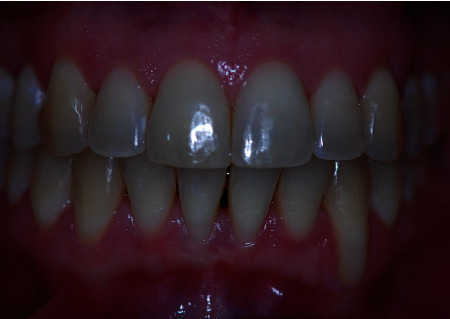
Preoperative front view of the patient showing a deep recession at #22.

A 28-year-old nonsmoking female was referred to a private practice in Londrina, Parana, Brazil, in February 2017 for the treatment of an isolated deep-wide recession [6] on the buccal aspect of tooth #22 (Figure 1). The patient presented in good general health with no contraindications to receiving surgical periodontal therapy. Periodontal examination revealed a normal biotype, 1 mm probing depth at the midfacial aspect, and a Miller's Class II marginal tissue recession [7] that was 7 mm in-depth and 5 mm wide.

All measurements were taken on the midbuccal aspect of the teeth. Measurements were taken with the University of North Carolina (UNC-15) probe with 1 mm markings and rounded to the nearest 0.5 mm. Baseline measurements (immediately before surgical treatment) were compared to measurements from the last follow-up appointment. Attached gingiva was determined by subtracting the pocket depth measurement from the apicocoronal dimension of the keratinized tissue. Marginal tissue recession was recorded as the distance from the cementoenamel junction to the tissue margin.

**Figure 2:**
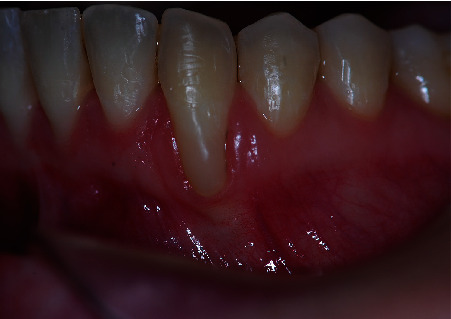
Preoperative close view showing the root defect covered with composite and an absence of keratinized tissue.

The first attempt to treat the area was using a composite on the root exposure to control her sensitivity (Figure 2). However, she was not satisfied with the esthetic appearance. Treatment options, including advantages and disadvantages, were discussed with the patient. Due to her financial limitations and great fear about surgical interventions, a two-step process including a combination of the modified apically repositioned flap (MARF) to increase the donor area adjacent to the defect and the laterally positioned flap (LPF) to cover the root recession was proposed and accepted by the patient. Oral informed consent was obtained before the treatment.

## 3. Case Management

Four main elements are required before the MARF technique is performed: (a) area free of inflammation, (b) no bone dehiscence, (c) minimal sulcus depth, and (d) ≤0.5 mm of attached gingiva. Before the incision was made, the level of crestal bone was probed to detect the presence of any bone dehiscence. For details, see Carnio and Miller [8].

**Figure 3:**
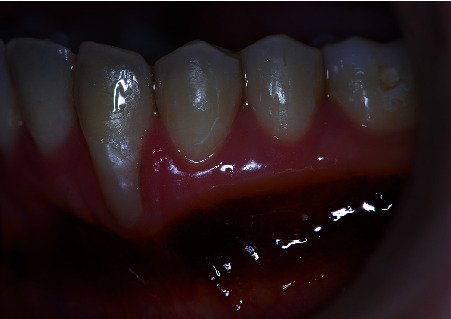
Iodine solution staining revealed the presence of approximately 3 mm of keratinized tissue at the donor area distal to the defect. This area was not adequate for an LPF.

The first step was to increase the donor area by using the MARF technique (Figure 3).

**Figure 4:**
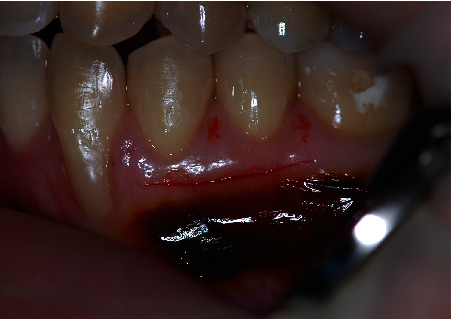
The MARF technique was used to increase the donor area. Initially, a horizontal incision was made onto the attached portion of the gingiva.

This consisted of a horizontal beveled incision made on the attached portion of keratinized tissue at an angle of 45 degrees, formed by the blade and the portion of the gingiva, making contact with periosteum at a point slightly apical to the alveolar crest (AC) (Figure 4). It is important to note that a beveled incision maintains part of connective tissue over the AC helping to protect it from reabsorption.

**Figure 5:**
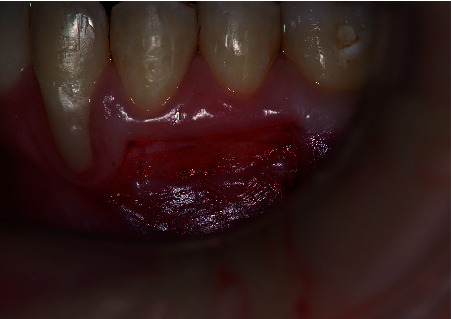
Next, two vertical incisions extending through the mucogingival line were made to connect to the horizontal incision. A split thickness was moved 3 mm apically and sutured.

The incision was extended from the mesial of tooth #23 to the distal of tooth #24. Two vertical incisions were then connected to the mesial and distal portions of the horizontal incision, extending beyond the mucogingival line. The split-thickness flap was repositioned 5 mm apically and secured with three single absorbable sutures (Figure 5). Periodontal dressing and sutures were removed after one week.

**Figure 6:**
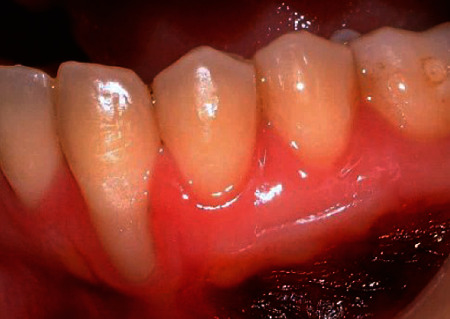
Eight weeks later, the donor area was increased to approximately 6 mm.

Eight weeks later (Figure 6), the second procedure was performed using the LPF. The LPF technique used the principle described by Grupe [9], in which a collar of marginal tissue is retained at the cervical area of the donor's tooth to avoid attachment loss, a common finding when this approach is not used [10].

**Figure 7:**
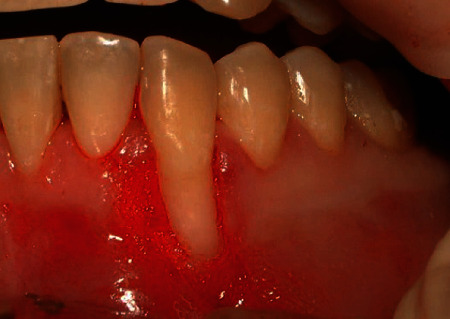
A beveled bleeding area was created mesially in the reception area.

**Figure 8:**
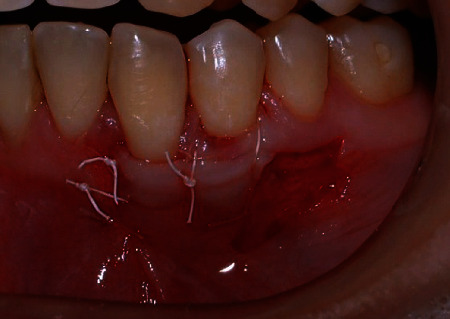
The 4.5 mm of keratinized tissue was moved laterally to the receptor area and sutured at CEJ. Note that the collar of 1.5 mm of keratinized tissue was maintained in its original position.

Initially, the composite was removed using a high-speed drill and the root was scaled and planed by curettes. Thereafter, a horizontal incision was performed on the donor area 1.5 mm apical to the gingival margin which was then connected by two vertical incisions as far as necessary to provide the flap mobility. A split-thickness flap with approximately 4.5 mm of keratinized tissue and free of tension was passively transferred laterally over the recipient area and sutured with nonabsorbable material [11] (Figures 7 and 8). The area was protected, and periodontal dressing and sutures were removed at one week. Both procedures healed uneventfully.

## 4. Clinical Outcomes

The result of the treated area using a combination of the MARF and LPF is summarized in Tables 1 and 2.

Table 1 shows the measurements of the donor area at baseline, eight weeks, and three years after performing the MARF technique. At eight weeks, the keratinized donor area showed a marked increase from 2.5 mm to 6 mm at tooth #23 and from 3.5 mm to 5.5 mm at tooth #24. Probing depth and marginal tissue recession remained stable during the course of observation.

**Figure 9:**
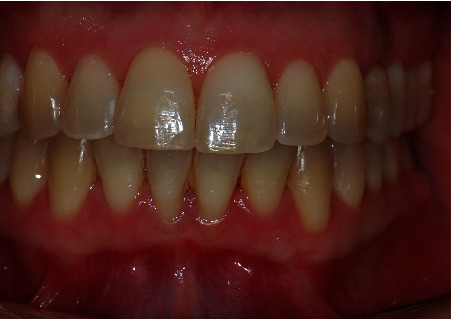
Postoperative front view at the three-year follow-up showing an excellent color match of the tissues.

**Figure 10:**
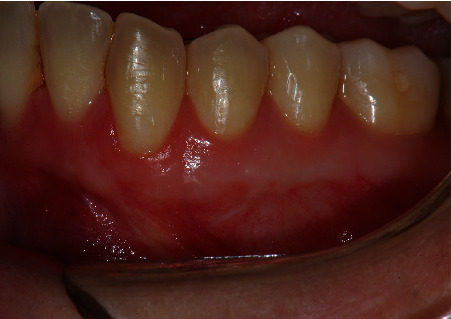
Close view of the treated area. Complete root coverage was obtained with 4 mm of keratinized tissue. Note that the donor area also increased its dimension with no additional recession.

At the recipient area, the recession decreased from 7 mm to 0.0 mm, and the keratinized tissue and the attached gingiva increased from 0.0 mm to 4 mm and from 0.0 mm to 2.5 mm, respectively, in the three-year observation (Figures 9 and 10). The patient reported an absence of sensitivity in the area.

## 5. Discussion

The LPF technique is a highly effective and predictable procedure to treat a single recession. It is simple to perform, produces ideal esthetic results, and does not require a palatal donor tissue. The main problem with this procedure is not related to the technique *per se* but is related to the amount of adjacent donor tissue available before the procedure and the consequences of using what exists. When the entire adjacent donor gingiva was transferred laterally to the recipient site [12], significant marginal tissue recession occurred associated with loss in the apicocoronal dimensions of keratinized tissue [10]. Consequently, an appropriated dimension in the donor area is crucial to performing the LPF [13].

The use of a free epithelized palatal graft for enhancing the donor gingival volume is a functional approach, although it often fails to produce an ideal color match. It also requires a wound to the palatal donor site resulting in greater postoperative discomfort to the patient [14]. Such disadvantages were not found when the MARF was used to increase the donor tissue. It not only produced a functional result but also promoted better esthetic results while minimizing morbidity by decreasing postoperative discomfort [15].

The donor area was increased by MARF to approximately 6 mm at eight weeks. This amount was enough to allow a sufficient volume of keratinized tissue to be moved laterally and another amount to be retained as a marginal tissue avoiding loss of attachment at the donor site. It is interesting to note that at the three-year follow-up, the donor areas still retained their increased volume when compared to the baseline (Table 1). This is in contrast with previous findings that demonstrated long-term loss of attachment and tissue volume at the donor area when LPF is performed [16].

The MARF technique was crucial to increase the feasibility of performing the LPF as a secondary procedure. By increasing the donor site, there was enough tissue to be partially transferred laterally and partially retained as a marginal collar. It provided the donor area the ability to heal with no additional recession and the recipient site with sufficient keratinized tissue to cover and to stabilize the recession in the long term.

Although the combination of these techniques presented in this report provided the complete resolution of the defect, it had a disadvantage of promoting two surgical procedures at a different time. Other available possibilities using a GTG in a single step may minimize this disadvantage [17, 18]. However, all the alternatives have in common the use of tissues adjacent to the defect to cover the exposed root. It is important to note that deep recessions usually have a lack of keratinized tissue apically as well as a shallow vestibule. That is the reason approaches using tissues adjacent to the recession are useful for the treatment of deep cases.

Even though two surgical steps were used, the combination of these simple methods could be an alternative in treating single defects due to the simplicity, no need for a palatal donor source, lower cost, excellent esthetic results, less morbidity, and better postoperative outcomes. Also, it resulted in an increased patient satisfaction since this process also decreased her anxiety about extended dental procedures [15, 19, 20].

## Figures and Tables

**Table 1:** Clinical buccal measures of the donor area at baseline, at eight weeks, and at three years.

Tooth #	Baseline	#23	3 years	Baseline	#24	3 years
Measurements		8 weeks			8 weeks	
PD	1	1	1.5	1	1	1.5
MTR	0.5	0.5	0	0	0.5	0
KT	2.5	6	5	3.5	5.5	5
AG	1.5	5	3.5	2.5	4.5	3.5

PD = probing depth; MTR = marginal tissue recession; KT = keratinized tissue; AG = attached gingiva.

**Table 2:** Clinical buccal measures of the recipient area at baseline and at three years.

Tooth #	#22	3 years
Measurements	Baseline	
PD	1	1.5
MTR	7	0
KT	0	4
AG	0	2.5

PD = probing depth; MTR = marginal tissue recession; KT = keratinized tissue; AG = attached gingiva.
